# Mature tertiary lymphoid structures support B cell-mediated antitumour immunity and are disrupted by neoadjuvant therapy in rectal cancer: a multicentre, retrospective study

**DOI:** 10.1016/j.ebiom.2025.106030

**Published:** 2025-11-19

**Authors:** Na Tian, Qianyu Wang, Yuan Lv, Wentao Zhong, Weixuan Li, Huiyun Cai, Ran An, Hongyan Zhu, Liang Sun, Qiang Yuan, Xing Dong, Junhua Dong, Junchao Bai, Aijun Liu, Gang Chen, Bin Wu, Junfeng Du

**Affiliations:** a Senior Department of General Surgery, The First Medical Centre, Chinese PLA General Hospital, Beijing, 100853, China; b Beijing Chest Hospital, Capital Medical University, Beijing, 101149, China; c Department of General Surgery, Peking Union Medical College Hospital, Chinese Academy of Medical Sciences & Peking Union Medical College, Beijing, 100730, China; d Department of General Surgery, The Seventh Medical Centre, Chinese PLA General Hospital, Beijing, 100700, China; e Department of Pathology, The Seventh Medical Centre, Chinese PLA General Hospital, Beijing, 100700, China

**Keywords:** Tertiary lymphoid structures, Neoadjuvant therapy, Tumour immune microenvironment, Rectal cancer

## Abstract

**Background:**

The tumour immune microenvironment (TIME), particularly the presence and maturation of tertiary lymphoid structures (TLS), shapes antitumour immunity and therapy response. However, the role of mature TLS (mTLS) in rectal cancer (RC) and their modulation by neoadjuvant therapy (neoTx) remain unclear.

**Methods:**

In this multicentre, retrospective study, we analysed patients with RC from two cohorts. Multi-omics profiling in patients with locally advanced rectal cancer (LARC) receiving no treatment (NT) included bulk RNA-seq (n = 123), immunohistochemistry and multiplex immunofluorescence (n = 161), scRNA-seq (n = 10) with paired scBCR-seq (n = 10). An independent neoTx cohort was used to assess treatment-induced immune changes, including bulk RNA-seq (n = 19) and immunohistochemistry (n = 125).

**Findings:**

mTLS tumours were characterised by plasma cells and CD8^+^ T cells being located in close spatial proximity to each other. B cell–related signatures—including plasma cells, germinal centre B cells, and follicular B cells—as well as CD138, IgG, and IgA expression were elevated in mTLS tumours. scRNA-seq and scBCR-seq analyses further revealed that mTLS tumours harboured a greater abundance of plasma cells, broader clonal diversity, and a higher proportion of IgG^+^ and IgA^+^ plasma cells. High CD138 expression correlated with favourable survival. Post-neoTx tumours showed higher CD4^+^, CD8^+^, and CD45RO^+^ T cell densities and lower mTLS presence. Notably, B cell gene signatures and CD20^+^ cell density were enriched in responders to neoTx, despite no difference in TLS maturation.

**Interpretation:**

mTLS are associated with enhanced B cell–mediated immune features and favourable prognosis. Post-neoTx is correlated with increased T cell infiltration but decreased TLS presence. The sustained B cell activation observed in non-responders raises the possibility that therapeutic strategies aimed at preserving or enhancing humoural immunity may benefit this patient subset.

**Funding:**

This study was supported by grants from the Natural Science Foundation of Beijing (7242034), New Technologies and Businesses of the PLAGH (5156ZE1X).

**Research in context**

**Evidence before this study**

TLSs are associated with improved prognosis and B cell–driven immunity in solid tumours. Emerging evidence suggests that mTLS support antibody production, but their role in LARC, particularly under neoTx, remains underexplored.

**Added value of this study**

We investigated the biological significance of TLS in locally advanced rectal cancer (LARC) using multi-omics approaches. mTLS tumours were enriched in plasma cells, exhibited elevated immunoglobulin expression, and were associated with favourable survival. Post-neoTx tumour was linked to increased T cell infiltration but reduced mTLS formation. Notably, higher enrichment of B cell–related features was observed in no-responders tumour, suggesting a potential role in therapy resistance, and underscoring their possible therapeutic relevance.

**Implications of all the available evidence**

This study highlights mTLS as critical hubs of antitumour immunity in LARC. NeoTx reshapes the TIME, potentially compromising B cell–mediated responses. Future strategies should consider preserving or enhancing TLS function, particularly in B cell–rich, treatment-resistant tumours.

## Introduction

Rectal cancer (RC) remains a major global health burden, particularly in its locally advanced rectal cancer (LARC), which is associated with high rates of recurrence and mortality.^1^ Despite advances in surgical techniques and systemic therapies, prognosis remains suboptimal. The tumour immune microenvironment (TIME) has emerged as a critical determinant of tumour progression and therapeutic response.^2^ Among its components, tertiary lymphoid structures (TLSs)—ectopic lymphoid aggregates resembling secondary lymphoid organs (SLOs)^3^—have attracted considerable attention for their role in orchestrating local antitumour immune responses.^4^, ^5^, ^6^ TLSs are composed of spatially organised T cells, B cells, follicular dendritic cells (FDCs), and high endothelial venules,^7^ and can be classified into immature and mature forms based on their degree of organisation and presence of germinal centre (GC).^8^, ^9^, ^10^ Recent studies suggest that mature TLS (mTLS) facilitate B cell activation and differentiation,^6^^,^^11^ particularly promoting plasma cell generation in renal cell carcinoma,^12^ and thereby contribute to effective humoural responses in various malignancies. However, the specific role of TLSs in LARC, particularly in orchestrating B-cell-mediated immune responses within the TIME, remains poorly defined.

In recent years, neoadjuvant therapy (neoTx),^13^ including preoperative chemotherapy, radiotherapy, or combined modalities such as chemoradiotherapy, has been increasingly utilised in LARC to downstage tumours,^14^ enhance surgical resectability^15^ and reduce local recurrence.^16^ In addition to its cytotoxic effects, neoTx exerts immunomodulatory actions, altering immune cell infiltration and phenotype.^17^^,^^18^ While it has been shown to enhance T-cell recruitment,^19^ neoTx may also impair TLS maturation,^20^ potentially limiting B-cell-centred immunity. However, the extent to which neoTx reshapes the TLS architecture and affects the interplay between B and T cells within the TIME remains unclear.

To address these questions, we conducted a comprehensive multi-omics analysis of a large multicentre LARC cohort, integrating immunohistochemistry (IHC), multiplex immunofluorescence (mIF), bulk RNA sequencing (RNAseq), and single-cell RNA sequencing (scRNA-seq) with paired single-cell B-cell receptor sequencing (scBCR-seq). This study focused on elucidating the role of mTLS in shaping antitumour immune responses. Additionally, we examined the impact of neoTx on the TIME, including its effects on TLS maturation, B-cell and T-cell dynamics, and the overall immune-regulatory landscape.

## Methods

### Ethics

The study was approved by the Institute Research Ethics Committees of the PLAGH (S2025-047-02) and PUMCH (I-25PJ0636).

### Patient population

Two independent clinical centres—PUMCH (Peking Union Medical College Hospital) and PLAGH (PLA General Hospital)—contributed patient samples for this study. The cohorts are referred to as the ‘PUMCH cohort’ and the ‘PLAGH cohort’. All patients provided written informed consent. For the no treatment (NT) cohort, IHC and mIF analyses (n = 161) were performed using tumour samples collected at PLAGH between January 2014 and January 2018. In addition, to gain deeper insight into the interaction between TLS and the TME, we analysed multi-omics data generated between April 2023 and August 2025 from both the PLAGH and PUMCH cohorts, including bulk RNA-seq (n = 123), scRNA-seq (n = 10), and paired scBCR-seq (n = 10). Furthermore, a neoTx cohort from both centres was included to evaluate the association between TLS status and treatment response, comprising bulk RNA-seq (n = 19) and IHC (n = 125).

For both scRNA-seq and bulk RNA-seq, tumour tissue samples were collected exclusively from the tumour centre. Corresponding haematoxylin and eosin (H&E)-stained sections were reviewed to confirm that tumour cells accounted for at least 90% of the tissue area before proceeding with sequencing. This criterion ensured high tumour purity in the sequenced samples.

### Clinicopathological characteristics

Clinicopathological features included age, sex, tumour stage, vascular invasion, perineural invasion and tumour grade. The TNM stage was reassessed according to the 8th edition of the American Joint Committee on Cancer (AJCC) guidelines. Sex information was recorded based on biological sex assigned at birth, as documented in the patients’ medical records. Data on race and ethnicity were not collected due to the homogeneous nature of the study cohort.

Overall survival (OS) was defined as the time from surgical treatment to death from any cause. We first reviewed haematoxylin and eosin (H&E)-stained sections to identify representative regions, then retrieved the corresponding formalin-fixed paraffin-embedded (FFPE) tissue blocks, from which new 4-μm-thick sections were cut for further analysis. These sections were subsequently used for IHC and mIF staining. All the representative H&E-stained sections were scanned using slide scanner (SQS-40 P, TEKSQRAY, China) and analysed using ImageViewer (DPVIEW, TEKSQRAY) software.

Tumour regression score (TRS) in the resected specimen after neoTx was calculated using the recommendations provided by College of American Pathologists and AJCC guidelines. Patients with TRS 0 and 1 were considered as responders, and those with TRS 2 and 3 as non-responders.

### RNA extraction and sequencing

For RNA-seq analysis, we performed a bulk RNA-seq analysis on fresh-frozen samples from patients with LARC. Total RNA was extracted from minced tissue specimens using Trizol reagent. The extracted RNA was purified utilising the Qiagen RNeasy Mini Kit and RNA quality and quantity were assessed using a NanoDrop and Agilent Bioanalyzer, respectively. First-strand cDNA synthesis was performed, and libraries were prepared using the Illumina TruSeq Stranded mRNA Library Prep Kit (Novogene®). The resulting libraries underwent sequencing on the Illumina platform for comprehensive RNA quality control and whole transcriptome sequencing analysis.

### IHC

Immunohistochemistry (IHC) was performed on 4-μm-thick FFPE sections following standard protocols.^20^ Tissue sections were deparaffinized, rehydrated, and incubated with 3% hydrogen peroxide to block endogenous peroxidase activity. Antigen retrieval was carried out using heat-induced epitope retrieval. Slides were then incubated with the following primary antibodies: anti-CD4 (ab133616, 1:500, Abcam, RRID: AB_2750883), anti-CD8 (ab101500, 1:100, Abcam, RRID: AB_10710024), anti-CD20 (ab9475, 1:50, Abcam, RRID: AB_307267), anti-CD45RO (ab23, 1:1000, Abcam, RRID: AB_449887), anti-FOXP3 (ab20034, 1:800, Abcam, RRID: AB_445284), anti-CD138 (ZA-0584, 1:1, ZSGB-BIO, RRID:AB_3096084), anti-IgG (10284-1-AP, 1:100, Proteintech, RRID: AB_2877729), and anti-IgA (60099-1-Ig, 1:160000, Proteintech, RRID: AB_1557448). Secondary antibodies included Goat Anti-Mouse IgG H&L (HRP) (ab6789, 1:1000, Abcam, RRID: AB_955439) and Goat Anti-Rabbit IgG H&L (HRP) (ab6721, 1:1000, Abcam, RRID: AB_955447). Signal detection was performed using 3,3′-diaminobenzidine (DAB), followed by haematoxylin counterstaining. Sections were dehydrated and mounted with neutral resin. Slides were scanned using a Leica CS2 system and analysed with Aperio ImageScope. To ensure technical reproducibility and minimise inter-batch variability, a randomly selected 10% subset of cases was re-stained and compared across staining runs, demonstrating high consistency in both staining intensity and marker localisation. Immune cell densities (CD4^+^, CD8^+^, CD20^+^, CD45RO^+^, FOXP3^+^, CD138^+^) within tumour regions were quantified using QuPath (v 0.5.1).^21^ For subsequent survival analyses, patients were stratified into high- and low-density groups based on the median value of each marker within the tumour region.

To ensure reproducibility and minimise inter-batch variability, all staining runs included appropriate positive and negative controls and were performed in technical duplicates. In addition, a randomly selected 10% subset of cases was re-stained across independent batches, which confirmed high concordance in both staining intensity and marker localisation. All stained slides were independently and blindly reviewed by two trained investigators, and discrepant cases were jointly re-evaluated to reach consensus.

### Multiplex immunofluorescence

mIF was performed by staining 4-um-thick FFPE whole tissue sections with standard, primary antibodies sequentially and paired with TSA 7-colour kit (abs50015-100 T, Absinbio), and stained with 4’,6-diamidino-2-phenylindole (DAPI). In brief, slides were deparaffinized, rehydrated, and subjected to repeated cycles of primary antibody incubation, HRP-conjugated secondary antibody binding (abs50015-02, Absinbio), fluorophore development (strictly timed at 10 min), microwave-based antigen stripping in citrate buffer (90 °C), and washing. To characterise spatial immune phenotypes, four staining panels were used: Panel 1: anti-CD20 (abs171788, 1:1000, Absinbio) and anti-CD23 (ab135386, 1:25, Abcam); Panel 2 anti-CD138 (ZA-0584, 1:1, ZSGB-BIO, RRID: AB_3096084) and anti-PANCK (abs123684, 1:400, Absinbio); Panel 3: anti-IgG (10284-1-AP, 1:200, Proteintech, RRID: AB_2877729) and anti-IgA (60099-1-Ig, 1:400, Proteintech, RRID: AB_1557448); Panel 4: anti-CD8 (66868-1-Ig, 1:5000, Proteintech, RRID: AB_2882205), anti-CD20 (abs171788, 1:1000, Absinbio), and anti-CD138 (ZA-0584, 1:1, ZSGB-BIO, RRID: AB_3096084). Slides were finally counterstained with DAPI (abs47047616; Absinbio), air-dried, manually coverslipped, and scanned using the Pannoramic MIDI II system (3DHISTECH).

Images were analysed using the Indica HALO software. Regions of mTLS were specifically selected, and nearest-neighbour analysis was applied to quantify the spatial proximity between CD8^+^ T cells and CD138^+^ plasma cells within these regions. The average nearest distance and standard deviation (SD) were calculated for mTLS. Detailed parameters and thresholds used in the analysis are provided in the Supplementary Materials.

The mIF assay was validated in accordance with the Society for Immunotherapy of Cancer (SITC) guidelines.^22^ Validation steps included assessment of antibody specificity, signal-to-noise ratio, reproducibility, and spectral separation to avoid fluorescence overlap. Each staining batch included appropriate positive and negative controls. Serial tumour sections were repeatedly stained to ensure consistency.

### Quantification of mature TLS

All cases were reviewed blindly by 2 pathologists (H.Y.Z, R.A.) for the presence and maturation of TLS according to the HE, the IHC and mIF stainings on serial sections. TLS were defined as rounded lymphoid aggregates composed of both B and T lymphocytes, located either intratumour or within 1 mm of the invasive margin, and comprising more than 50 lymphocytes. To ensure reproducibility, the maturation status of TLS was classified according to the proportion of CD23^+^ FDCs within the TLS area. Specifically, mTLS were defined as structures with well-organised GC containing CD23^+^ FDC networks occupying more than 10% of the TLS area, whereas TLSs lacking CD23 expression or showing CD23^+^ FDC coverage of less than 10% were classified as immature TLS (iTLS). The 10% threshold was determined based on the distribution pattern of CD23^+^ area ratios across all TLSs, which clearly separated poorly organised from well-structured TLSs.

The reproducibility to assess the TLS status of tumour samples with the pathology method was tested by training a third senior pathologist (A.J.L) for TLS screening on a set of 15 samples. The trained pathologist subsequently analysed 161 tumour samples. The Cohen's kappa coefficient (κ) test was used to measure inter-rater reliability for the following criteria: 1) presence of TLS and 2) maturity of TLS. The inter-rater agreement rate was 0.814 (Cohen's Kappa score, CI 95: 0.608–1.0) for the presence of TLS and 0.891 to evaluate the maturity of the TLS (range 0.813–0.970).

### scRNA-Seq workflow

Tissues were transported in sterile culture dishes with 10 mL 1 × Dulbecco's Phosphate-Buffered Saline (DPBS; Thermo Fisher) on ice to remove the residual tissue storage solution, and minced on ice. We used dissociation enzyme 0.25% Trypsin (Thermo Fisher) and 10 μg/mL DNase I (Sigma) dissolved in PBS with 5% fetal bovine serum (FBS; Thermo Fisher) to digest the tissues. Tissues were dissociated at 37 °C with a shaking speed of 50 rpm for about 40 min. We repeatedly collected the dissociated cells at 20-min intervals to increase cell yield and viability. Cell suspensions were filtered using a 40-μm nylon cell strainer and red blood cells were removed by 1 × Red Blood Cell Lysis Solution (Thermo Fisher). Dissociated cells were washed with 1 × DPBS containing 2% FBS. Cells were stained with 0.4% Trypan Blue (Thermo Fisher) to check the viability on a Countess® II Automated Cell Counter (Thermo Fisher).

### 10× library preparation and sequencing

Beads with unique molecular identifiers (UMIs) and cell barcodes were loaded close to saturation so that each cell was paired with a bead in a Gel Beads-in-Emulsion. After exposure to cell lysis buffer, polyadenylated RNA molecules hybridised to the beads. Beads were retrieved into a single tube for reverse transcription. On cDNA synthesis, each cDNA molecule was tagged on the 5′ end with a UMI and cell label indicating its cell of origin. 10× beads were subjected to second-strand cDNA synthesis, adaptor ligation and universal amplification. Sequencing libraries were prepared using randomly interrupted whole-transcriptome amplification products to enrich the 5′ end of the transcripts linked with the cell barcode and UMI. Single-cell RNA libraries were prepared using the Chromium Single Cell 5′ v2 Reagent (10× Genomics), and Chromium Single Cell VDJ Reagent Kits (10× Genomics) were used to prepare single-cell RNA libraries. Each sequencing library was generated with a unique sample index. Sequencing libraries were quantified using a High Sensitivity DNA Chip (Agilent) on a Bioanalyzer 2100 and the Qubit High Sensitivity DNA Assay (Thermo Fisher Scientific). The libraries were sequenced on Xplus (Illumina/MGI DNBSEQ-T7) using 2 × 150 chemistry.

### scRNA/BCR-seq data processing

scRNA-seq and scBCR-seq were assembled and quantified using Cell Ranger (v9.0.1) multiprotocol against the GRCh38 reference genome. Assembled contigs labelled as low-confidence, nonproductive, or with UMIs <2 were discarded. Only cells with at least one heavy chain (IGH) and one light chain (IGL or IGK) were retained. For a given B cell, if two or more IGH or IGL/IGK sequences were assembled, the sequence with the highest expression level (UMI or reads) was defined as the dominant IGH or IGL/IGK. Each unique dominant IGH–IGL/IGK pair (including CDR3 amino acid sequences and rearranged VDJ genes) was defined as a clonotype.

### scRNA-seq data analysis

scRNA-seq data generated by the 10× Genomics platform were processed by aligning raw reads to the human genome (GRCh38) using Cell Ranger (v9.0.1), generating a feature-barcode matrix. Low-quality cells (those with <300 genes, >7000 genes, >20% mitochondrial content or >3% red blood cell genes content) were filtered out to ensure data integrity. Additionally, DoubletFinder R package (v2.0.4) was applied to identify and remove potential doublets based on cell clustering and artificial nearest neighbour generation.^23^ The expected doublet rate was set to be 0.08 and cells with doubletScore >0.25 were regarded as doublets and filtered out.

Data normalisation was performed using the Seurat R package (v5.2.1), where the NormalizeData and ScaleData functions were applied to minimise technical noise. Harmony R package (v1.2.3) was used to correct batch effects.^24^ Principal component analysis (PCA) was applied for dimensionality reduction, and the top 50 principal components were selected for downstream analyses. Unsupervised clustering was performed using the FindNeighbors and FindClusters functions. Visualisation of the clustering results was achieved through UMAP or TSNE. Differential gene expression analysis was conducted using the FindAllMarkers function, and cell populations were annotated using a combination of classical marker genes and those from published literature. The cluster with multiple well-defined marker genes of different cell types was considered cell contamination and removed in downstream analysis.

### scBCR prediction

The resulting single-cell transcriptomic data and VDJ data were integrated into the R environment. Using the scRepertoire R package (v2.3.4) package, the combineBCR functions aggregated BCR sequences by cell barcode, allowing for the identification of clonotypes.^25^ These clonotypes were then integrated with scRNA-seq data for further analysis and visualisation using Seurat. Based on IGH gene expression characteristics, plasma cells expressing IGH were further classified into IgG^+^, IgA^+^, IgD^+^, and IgM^+^ plasma cells.

### GO and KEGG pathway enrichment analysis

Differentially expressed genes were identified using the DESeq2 R package (v1.46.0), with an adjusted P value < 0.05 and |log2 fold change| > 1 as the threshold. Gene Ontology (GO) classification—including biological process (BP), molecular function (MF), and cellular component (CC)—was performed to explore the potential functions of differentially expressed genes (DEGs) and reveal biological relevance. Kyoto Encyclopedia of Genes and Genomes (KEGG) pathway analysis was conducted to further investigate the enriched signalling pathways. Both GO and KEGG analyses were carried out using the clusterProfiler R package (v3.8). Enrichment was considered significant if the nominal P value was <0.05 and the false discovery rate (FDR) q-value was <0.25.

### Immune cell infiltration estimation

To assess the reliable results of immune score evaluation, we used immunedeconv R package (v 3.8), including four latest algorithms (CIBERSORT, XCELL, MCPCOUNTER and QUANTISEQ). CIBERSORT estimates relative immune cell fractions within each sample rather than absolute cell counts. Samples with missing values were excluded, and only those with P < 0.05 were considered for further analysis.

### Analysis of public transcriptomic data

To validate the prognostic value of key gene signatures identified in our cohort, we utilised The Cancer Genome Atlas Rectal Adenocarcinoma (TCGA-READ) dataset through the GEPIA3 online platform^26^ (https://gepia3.bioinfoliu.com/). OS analysis was performed using the default settings. For the plasma cell signature, a 14-gene set comprising (including *MZB1*, *DERL3*, *JSRP1*, *TNFRSF17*, *SLAMF7*, *IGHG1*, *IGHGP*, *IGLV3-1*, *IGLV6-57*, *IGHA2*, *IGKV4-1*, *IGKV1-12*, *IGLC7*, and *IGLL5*) was used. TLS maturation was proxied by assessing CXCL13 expression level.

### Statistical analysis

The statistical analysis software used in this study was R version 4.4.2, and GraphPad Prism version 8. Mann–Whitney tests were used to compare continuous variables. Chi-square or Fisher's exact tests were used to compare categorical variables. OS was compared using the Kaplan–Meier and log-rank survival analysis. Hazard ratios (HRs) and 95% confidence intervals (CIs) were calculated using Cox regression analysis. All statistical tests were two-sided, and P < 0.05 was considered statistically significant.

### Role of the funding source

The funders had no role in paper design, data collection, data analysis, interpretation and writing of the paper.

## Results

### Plasma cells preferentially accumulate within mTLS in rectal cancer

**Fig. 1:**
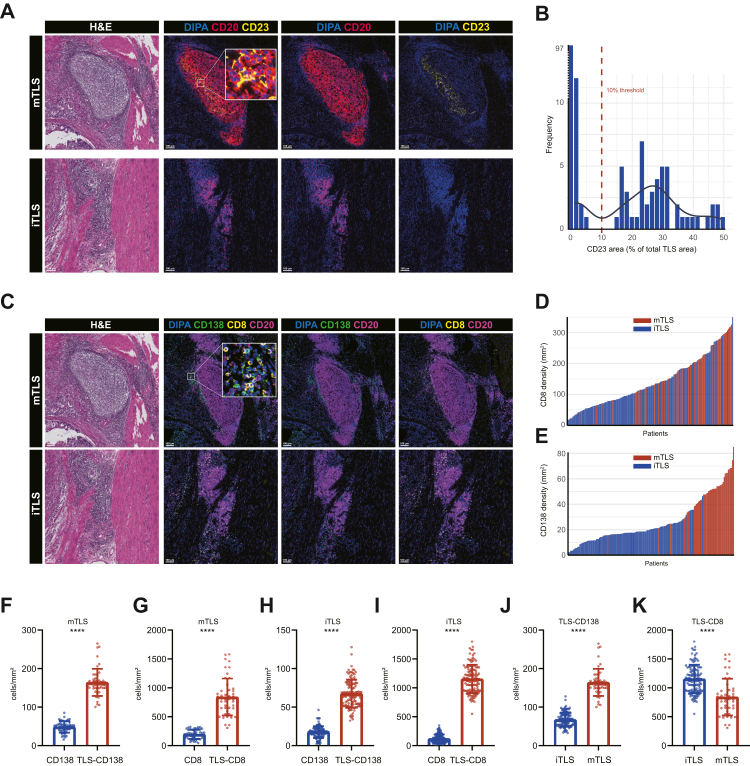
**H&E staining and mIF for the indicated markers for representative mTLS and iTLS groups**. (A) Cellular phenotypes displayed were CD20^+^ (B cells), and CD20^+^/CD23^+^ (GC B cells). Scale bar indicates 100 μm. (B) Distribution of CD23^+^ FDC area within total TLS regions. (C) Cellular phenotypes displayed were CD138^+^ (plasma cells), CD8^+^ (CD8T cells), CD20^+^ (B cells). (D–E) Distribution of CD8^+^ and CD138^+^ cell densities across TLS maturation states. (F–G) Correlations between TLS-localised and total tumour CD138^+^ or CD8^+^ cell densities in mTLS group. P values were derived from the Mann–Whitney U test. (H–I) Correlations between TLS-localised and total tumour CD138^+^ or CD8^+^ cell densities in iTLS group. P values were derived from the Mann–Whitney U test. (J–K) Correlations of CD138^+^ and CD8^+^ cell densities between iTLS and mTLS regions. P values were derived from the Mann–Whitney U test. Scale bar indicates 100 μm ∗P < 0.05, ∗∗P < 0.01, ∗∗∗P < 0.001, ∗∗∗∗P < 0.0001.

In our previous work, we demonstrated that B cells were predominantly found in tumours with abundant TLS.^20^ To further investigate the role of TLSs, we evaluated tumour samples from 161 patients with LARC in NT cohort. H&E and mIF staining were used to assess TLS presence and maturation. Samples were classified into mTLS and iTLS groups (Fig. 1A). We assessed the distribution of CD23^+^ FDC area of total TLS area (Fig. 1B). The detailed clinicopathological characteristics of the NT cohorts are presented in Table 1. In the NT cohort, 48 patients (29.8%) were classified as mTLS, while 113 patients (70.2%) were classified as iTLS. There were no significant differences between the mTLS and iTLS groups in terms of age, sex, TNM stage, vascular invasion, perineural invasion, or tumour grade. In mTLS tumours, CD138^+^ plasma cells and CD8^+^ T cells were primarily located around the GC within the mTLS area, whereas in iTLS tumours, they were more diffusely distributed throughout the iTLS area (Fig. 1C). We assessed the distribution of CD8^+^ density and TLS maturation (Fig. 1D), and the distribution of CD138^+^ plasma cell density and TLS maturation (Fig. 1E). Quantitative analysis revealed that the densities of CD8^+^ T cell and CD138^+^ plasma cell were significantly higher inside TLS compared with tumour regions in mTLS group (all P < 0.0001, Mann–Whitney U test; Fig. 1F and G). A comparable finding was observed in the iTLS group (all P < 0.0001, Mann–Whitney U tests; Fig. 1H and I). Comparative analysis of TLS subtypes showed that the density of CD138^+^ plasma cell was significantly greater within mTLS than within iTLS (P < 0.0001, Mann–Whitney U tests; Fig. 1J), whereas the density of CD8^+^ T cell was lower within mTLS than within iTLS (P < 0.0001, Mann–Whitney U test; Fig. 1K). These data indicate that while both TLS subtypes are associated with immune cell accumulation, mTLS preferentially enrich for plasma cells, whereas iTLS are more closely associated with CD8^+^ T cell infiltration. In addition, mIF further revealed that the CD8^+^ T cell area closely overlapped (29.02 ± 26.64 μm) with CD138^+^ plasma cell area, suggesting potential functional interactions between these two immune subsets.

**Table 1:** Characteristics of neoTx and NT groups (N = 312).

Clinicopathological characteristics	NT No. (%) (n = 161)	neoTx No. (%) (n = 151)	P^a^
Median age (IQR), years	57.65 ± 10.14	55.87 ± 11.87	0.156
Age			0.675
≤60	89 (55.3%)	88 (58.3%)	
>60	72 (44.7%)	63 (41.7%)	
Sex			0.565
Male	117 (72.7%)	115 (76.2%)	
Female	44 (27.3%)	36 (23.8%)	
T stage			<0.001
T0	0 (0.0%)	26 (17.2%)	
T1	0 (0.0%)	1 (0.7%)	
T2	3 (1.9%)	28 (18.5%)	
T3	145 (90.1%)	87 (57.6%)	
T4	13 (8.1%)	9 (6.0%)	
N stage			0.37
N0	90 (55.9%)	96 (63.6%)	
N1	57 (35.4%)	43 (28.5%)	
N2	14 (8.7%)	12 (7.9%)	
TNM stage			<0.001
Stage 0	0 (0.0%)	26 (17.2%)	
Stage 1	0 (0.0%)	28 (18.5%)	
Stage 2	90 (55.9%)	42 (27.8%)	
Stage 3	71 (44.1%)	55 (36.4%)	
Lymphovascular invasion			0.284
Positive	126 (78.3%)	105 (84.0%)	
Negative	35 (21.7%)	20 (16.0%)	
Perineural invasion			0.642
Positive	128 (79.5%)	103 (82.4%)	
Negative	33 (20.5%)	22 (17.6%)	
Differentiation grade			<0.001
Low	21 (13.0%)	5 (4.0%)	
Medium	131 (81.4%)	98 (78.4%)	
High	9 (5.6%)	22 (17.6%)	
Recurrence			
Yes	50 (31.1%)	49 (32.5%)	0.886
No	111 (68.9%)	102 (67.5%)	

a P values derived from Fisher's exact test or χ^2^ test.

### Bulk RNA-seq reveals plasma cells enrichment in mTLS tumour

**Fig. 2:**
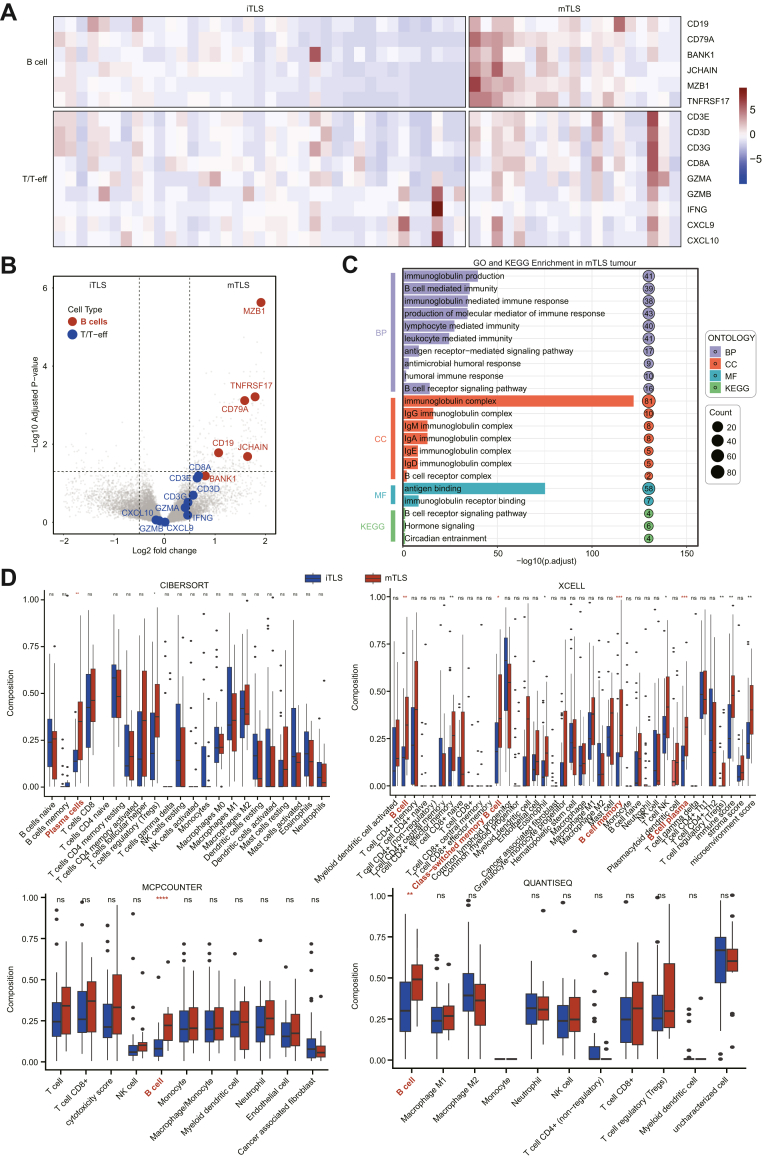
**mTLS tumours enriched for B-cell gene scores in the PLAGH cohort**. (A) Hierarchical cluster of the T and B cell subset signatures. Samples are ordered by mTLS and iTLS (n = 56). (B) Volcano plot representing differentially expressed genes between tumours with mTLS and iTLS. Genes from the T and B cell subset signatures are highlighted. (C) GO and KEGG enrichment analysis between the tumour with mTLS and iTLS. (D) Immune cell infiltration differences between mTLS and iTLS tumours were evaluated using computational tools, including CIBERSORT, XCELL, MCPCOUNTER, and QUANTISEQ. P values were derived from the Mann–Whitney U test. ∗P < 0.05, ∗∗P < 0.01, ∗∗∗P < 0.001, ∗∗∗∗P < 0.0001.

Differential gene expression analysis between the mTLS and iTLS groups in PLAGH cohort (n = 56), identifying 70 upregulated and 230 downregulated genes in the mTLS group. Considering that TLS primarily consist of T and B cell aggregates, we specifically examined transcriptional differences in these two cell populations. Heatmaps depicting gene expression patterns of T and B cells in the two groups are presented in Fig. 2A. Differential expression analysis revealed a significant enrichment of B-cell-associated genes in the mTLS group, whereas T-cell-related genes exhibited no notable enrichment (Fig. 2B). To gain deeper insights into the biological implications of these findings, we performed KEGG and GO enrichment analyses (Fig. 2C), revealing that the mTLS group was predominantly enriched in pathways associated with humoural immunity and B cell activation. Additionally, four widely used immune cell quantification algorithms (CIBERSORT, XCELL, MCPCOUNTER and QUANTISEQ) were applied to compare the immune cell composition between the two groups We found that mTLS tumours exhibited higher enrichment of B cells and plasma cells compared with iTLS tumours (Fig. 2D). Similar results were observed for PUMCH cohort (n = 67) (Supplementary Figure S1A–D). These algorithms revealed a strong association between mTLS and B-cell-mediated immune responses.

**Fig. 3:**
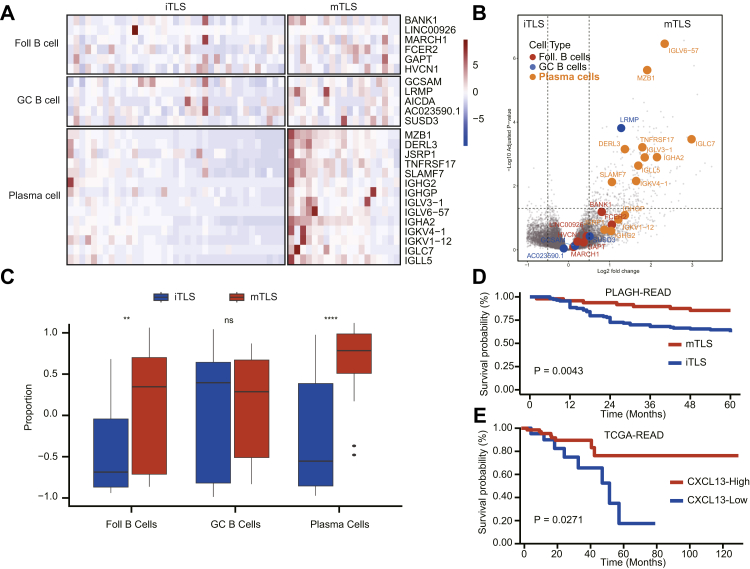
**mTLS tumours enriched for plasma-cell gene scores in the PLAGH cohort**. (A) Hierarchical cluster of the three B-cell subset signatures. Samples ordered by mTLS and iTLS (n = 56). (B) Volcano plot representing differentially expressed genes between tumours with mTLS and iTLS. Genes from the three B-cell signatures are highlighted. (C) Boxplots depicting signature Z scores for the plasma cells, GC B cells, and follicular B cells, grouped by mTLS and iTLS status. P values were derived from the Mann–Whitney U test. (D) OS was compared in tumours with mTLS and iTLS by Kaplan–Meier survival curve in PLAGH-READ cohort. (E) OS was compared in tumours with CXCL13-high and CXCL13-low by Kaplan–Meier survival curve in TCGA-READ cohort. P values of were calculated by log-rank test. ∗P < 0.05, ∗∗P < 0.01, ∗∗∗P < 0.001, ∗∗∗∗P < 0.0001.

We further examined the impact of mTLS on three distinct intratumoural B-cell subpopulations. Gene enrichment heatmaps comparing the two groups are shown in Fig. 3A. Differential expression analysis revealed a significant enrichment of plasma-cell-related genes in the mTLS group, with pronounced upregulation of genes such as MZB1, TNFRSF17, and various immunoglobulin genes (Fig. 3B). Additional enrichment analysis confirmed a significant increase in plasma cells and follicular B cells in the mTLS group (all P < 0.0001, Mann–Whitney U tests), whereas GC B cells showed no significant difference (Fig. 3C). Again, similar results were observed for PUMCH cohort (Supplementary Figure S2A–C). The mTLS group was significantly associated with improved OS compared with iTLS group in PLAGH-READ (P = 0.0043, log-rank test) (Fig. 3D). Given that CXCL13 is a key marker of TLS formation,^27^^,^^28^ we next evaluated its prognostic value in the TCGA-READ cohort. Consistent with our findings, high CXCL13 expression was correlated with favourable OS (P = 0.0271, log-rank test, Fig. 3E).

### Single-cell and BCR sequencing reveal functional and clonal diversity of plasma cells in mTLS tumours

**Fig. 4:**
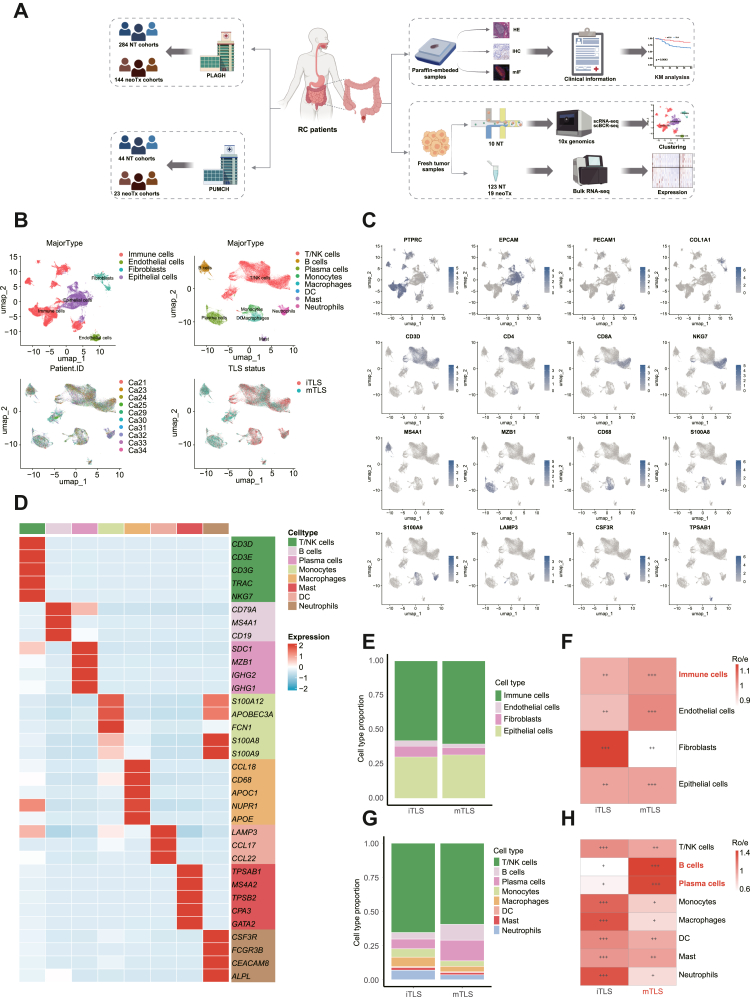
**scRNA-seq profiling of the landscape of LARC**. (A) Overview of the experimental design. (B) UMAP plot of 28 221 cells from LARC primary tumour samples; four and 11 main cell types were identified and colour-coded by cell type, patient ID and TLS status (n = 10). (C) Identification of various cell types based on expression of specified marker genes. (D) The accompanying heatmap highlights the expression of characteristic genes within these cell types. (E–F) Characterisation of the proportion of cell types identified in each sample in mTLS vs. iTLS tumours. (G–H) Characterisation of the proportion of immune cell types identified in each sample in mTLS vs. iTLS LARC tissue.

To further explore mTLS-related differences in the LARC TIME, we performed scRNA-seq on 10 patients with LARC (5 mTLS and 5 iTLS, Fig. 4A). After quality control, 105473 cells were retained (54427 from mTLS and 51046 from iTLS tumours). Unsupervised clustering identified four major cell types: immune cells, epithelial cells, endothelial cells and fibroblasts (Fig. 4B). Subclustering revealed finer immune subsets, including T/NK cells, B cells, plasma cells, monocytes, macrophages, DC, mast, and neutrophils (Fig. 4B–D). Compared with iTLS tumours, mTLS tumours exhibited higher proportions of immune cells, endothelial cells, and epithelial cells, but a significantly lower proportion of fibroblasts (Fig. 4E and F). iTLS tumours had significantly higher proportions of T/NK cells, monocytes, macrophages, DC, mast, and neutrophils, while mTLS tumours had increased B cells and plasma cells (Fig. 4G and H).

**Fig. 5:**
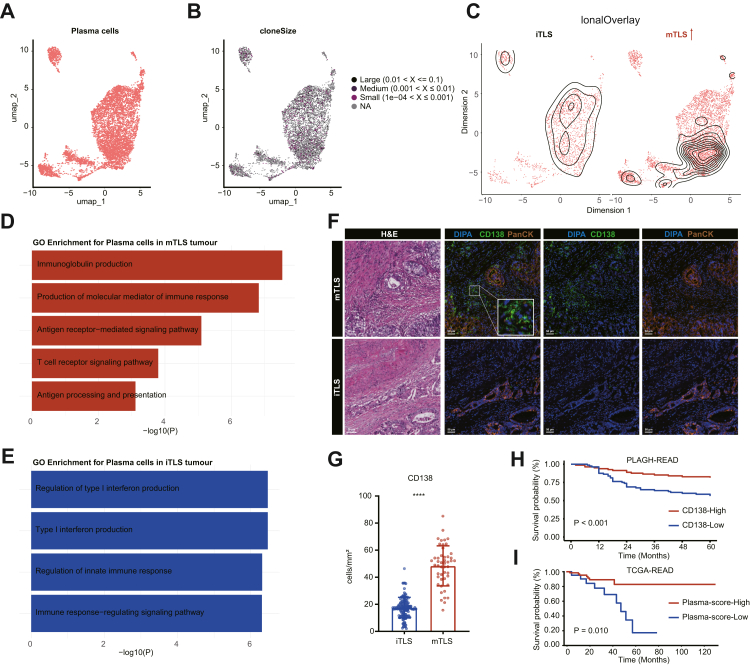
**scBCR-seq profiling reveals clonal diversity and functional features of plasma cells in LARC**. (A, B) Overview of the scBCR-seq analysis strategy for plasma cells (n = 10). (C) Comparison of plasma cell clonal diversity between mTLS and iTLS LARC tissues. (D, E) GO enrichment analysis in plasma cells from mTLS and iTLS tumours. (F) Representative mIF images showing CD138 expression in mTLS and iTLS tumours. Scale bar indicates 50 μm. (G) Quantification of CD138 expression levels in mTLS vs. iTLS tumours. P values were derived from the Mann–Whitney U test. (H) OS was compared in tumours with CD138-high and CD138-low by Kaplan–Meier survival curve in PLAGH-READ cohort. (I) OS was compared in tumours with CD138-score-high and CD138-score-low by Kaplan–Meier survival curve in TCGA-READ cohort. P values of were calculated by log rank test. ∗P < 0.05, ∗∗P < 0.01, ∗∗∗P < 0.001, ∗∗∗∗P < 0.0001.

To investigate the clonal characteristics of plasma cells, we performed scBCR-seq. scBCR-seq analysis revealed mTLS tumours displayed broader clonal diversity among plasma cells compared with iTLS tumours (Fig. 5A–C). GO enrichment analysis revealed that plasma cells in mTLS tumours exhibited stronger immunoglobulin production pathways, whereas those from iTLS tumours showed preferential enrichment of type I interferon pathways (Fig. 5D and E). Consistently, mIF confirmed that mTLS tumours exhibited significantly higher CD138 expression compared with iTLS tumours (P < 0.001, Mann–Whitney U tests) (Fig. 5F and G). High CD138 expression was associated with significantly improved OS compared with low CD138 expression (P < 0.001, log-rank test, Fig. 5H). Further, multivariate Cox regression analysis indicated that low CD138 expression was a significant adverse prognostic factor for OS (HR: 2.93, 95% CI: 1.55–5.54, P = 0.001, Supplementary Table S1). Consistent with our findings, high plasma-score expression was correlated with favourable OS (P = 0.010, log-rank test, Fig. 5I).

**Fig. 6:**
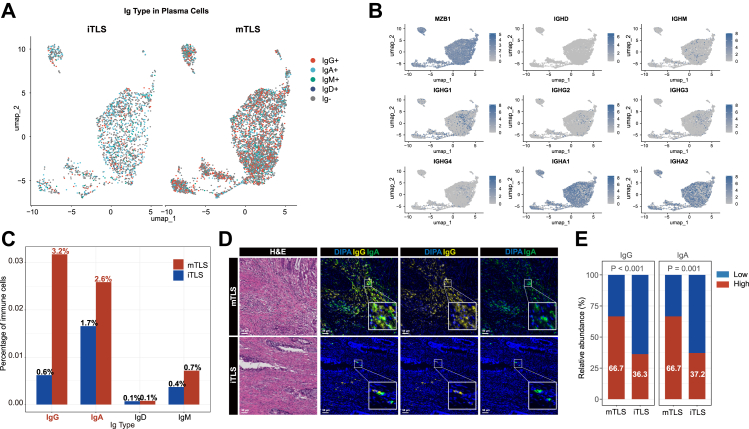
**Characterisation of immunoglobulin subtypes of plasma cells in LARC**. (A) Classification of plasma cells into IgG^+^, IgA^+^, IgM^+^ and IgD^+^ subtypes based on scBCR-seq data (n = 10). (B) Expression levels of key plasma cell marker genes (*MZB1*, *IGHD*, *IGHM*, *IGHG1*, *IGHG2*, *IGHG3*, *IGHG4*, *IGHA1* and *IGHA2*). (C) Proportions of IgG^+^ and IgA^+^ plasma cells in mTLS vs. iTLS tumours. (D–E) Representative mIF images showing IgG and IgA expression in mTLS and iTLS tumours. Scale bar indicates 50 μm.

Given that plasma cells exert their antitumour functions primarily through the secretion of specific antibodies, we further classified plasma cells into distinct immunoglobulin subtypes based on scBCR-seq data. These subtypes included IgG^+^, IgA^+^, IgM^+^ and IgD^+^ plasma cells (Fig. 6A). In LARC, we observed that the expression level of IGHG (encoding IgG heavy chains) in plasma cells was lower than that of IGHA (encoding IgA heavy chains) (Fig. 6B). However, the proportions of both IgG^+^ and IgA^+^ plasma cells among total immune cells were significantly higher in mTLS tumours than in iTLS tumours (Fig. 6C). Consistently, mIF analysis confirmed these findings, showing that protein levels of both IgG and IgA were significantly higher in mTLS tumours compared with iTLS tumours (Fig. 6D and E).

### NeoTx is associated with T-cell–dominated antitumour immunity independent of plasma cells

**Fig. 7:**
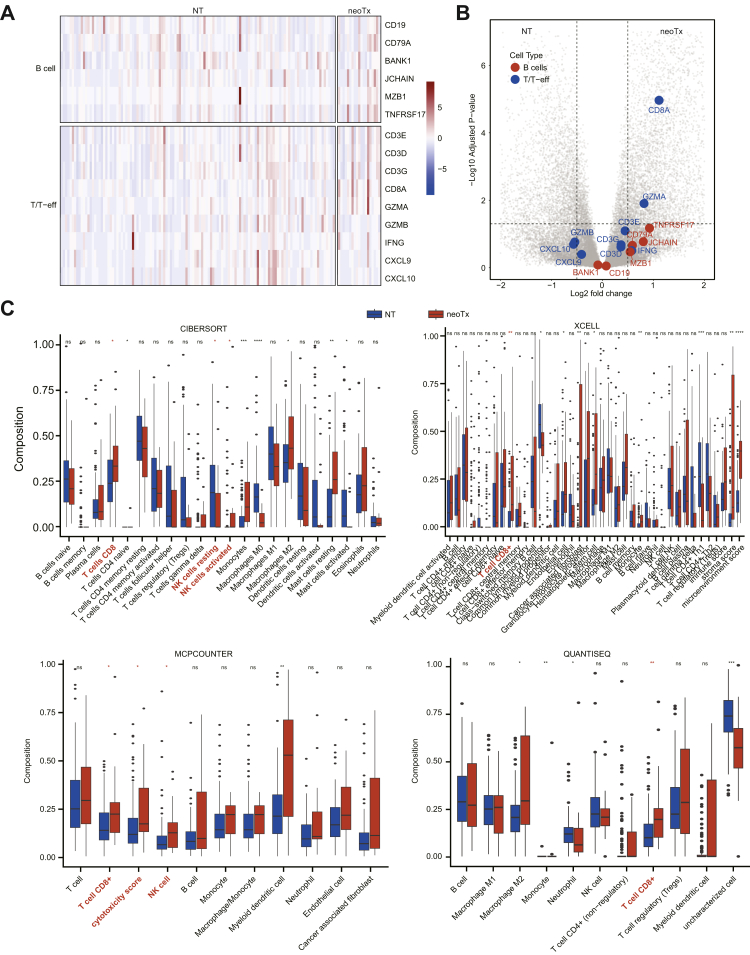
**Post-neoTx tumours are enriched for T-cell gene scores**. (A) Hierarchical cluster of the T and B cell subset signatures. Samples are ordered by neoTx (n = 19) and NT (n = 123). (B) Volcano plot representing differentially expressed genes between tumours with neoTx and NT. Genes from the T and B cell subset signatures are highlighted. (C) Immune cell infiltration differences between neoTx and NT tumours were evaluated using computational tools, including CIBERSORT, XCELL, MCPCOUNTER and QUANTISEQ. P values were derived from the Mann–Whitney U tests. ∗P < 0.05, ∗∗P < 0.01, ∗∗∗P < 0.001, ∗∗∗∗P < 0.0001.

Our previous studies have shown that neoTx was associated with a reduction in TLS density and maturation.^20^ To further investigate the effect of neoTx on plasma cells, we analysed bulk RNA-seq data from 19 neoTx and 123 NT tumours, examining the expression levels of B-cell- and T-cell-associated genes in both groups. While no significant differences were observed in B-cell-associated genes between the two groups, we detected a notable enrichment of cytotoxic T-cell-related genes, including CD3E, CD8A, and GZMA, in the neoTx group (Fig. 7A and B). We used four widely used immune cell quantification algorithms to compare immune cell composition between the two groups (Fig. 7C). The results consistently showed that the neoTx group was strongly associated with T-cell-mediated immune responses. These findings suggest that neoTx can activate a T-cell-mediated cytotoxic immune environment.

**Fig. 8:**
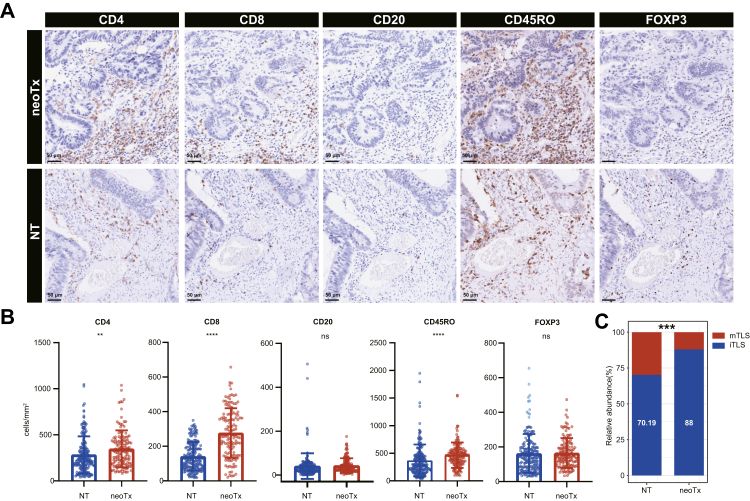
**Post-neoTx tumours are enriched for T cells**. (A) IHC staining for the indicated markers for representative neoTx (n = 125) and NT (n = 161). Scale bar indicates 50 μm. (B) Box plots depict the density of CD4^+^ T cells, CD 8^+^ T cells, CD20^+^ B cells, CD45RO^+^ T cells and Foxp3^+^ (Treg cells) grouped by neoTx and NT groups. Wilcoxon P values are reported. P values were derived from the Mann–Whitney U tests. (C) The expression ratio of mTLS in specimens was compared in neoTx and NT groups. Analysis was performed by Fisher's exact test. P values were derived from the χ2 test. ∗P < 0.05, ∗∗P < 0.01, ∗∗∗P < 0.001, ∗∗∗∗P < 0.0001.

To validate these findings, a total of 125 patients who received neoTx were evaluated (Supplementary Table S2). No significant differences in age or sex were observed between the NT and neoTx groups (Supplementary Table S2). IHC analysis revealed that, compared with NT group, the neoTx group exhibited significantly increased densities of CD8^+^ (P < 0.0001, Mann–Whitney U test), CD4^+^ (P < 0.01, Mann–Whitney U test), and CD45RO^+^ T cell (P < 0.0001, Mann–Whitney U test) (Fig. 8A and B). In contrast, the densities of CD20^+^ B cell and FOXP3^+^ Treg cell showed no significant differences between the neoTx and NT groups (P > 0.05, Mann–Whitney U tests) (Fig. 8A and B). These findings suggest that neoTx predominantly enhances T-cell-mediated antitumour immunity. Additionally, TLS maturation was significantly reduced in the neoTx group compared with the NT group (P < 0.001, Pearson's chi-square test) (Fig. 8C).

### B-cell-associated immune features are enriched in non-responders to neoTx

**Fig. 9:**
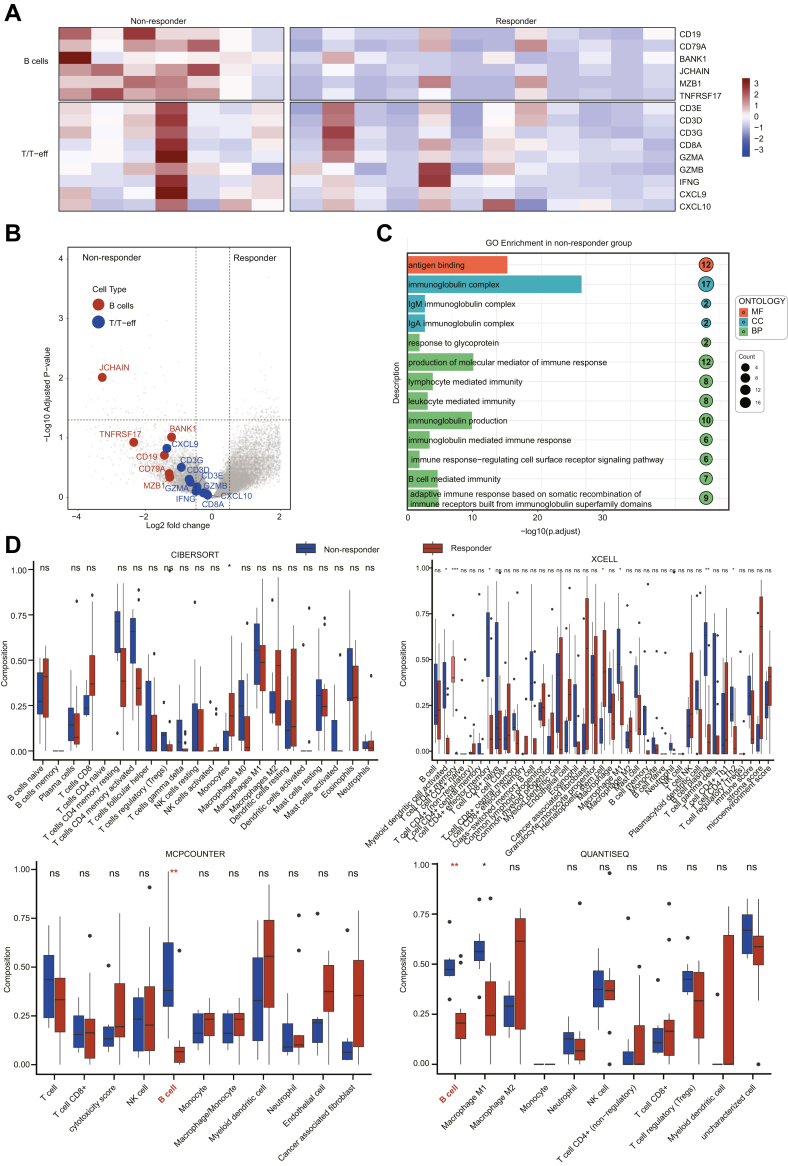
**Non-responder tumours are enriched for B-cell gene scores**. (A) Hierarchical cluster of the T and B cell subset signatures. Samples are ordered by responder and non-responder (n = 19). (B) Volcano plot representing differentially expressed genes between tumour with responder and non-responder. Genes from the T and B cell subset signatures are highlighted. (C) GO and KEGG enrichment analysis between the tumour with responder and non-responder. (D) Immune cell infiltration differences between responder and non-responder tumours were evaluated using computational tools, including CIBERSORT, XCELL, MCP, and QUANTISEQ. P values were derived from the Mann–Whitney U tests. ∗P < 0.05, ∗∗P < 0.01, ∗∗∗P < 0.001, ∗∗∗∗P < 0.0001.

We stratified patients in the neoTx group into responder and non-responder groups and performed differential gene expression analysis (Fig. 9A). No significant difference in the enrichment of cytotoxic T-cell or interferon signalling–related genes (*CD3E, CD3D, CD3G, CD8A, GZMA, GZMB, IFNG, CXCL9, and CXCL10*) was observed between the responder and no-responder groups, whereas B-cell–related gene enrichment (*JCHAIN*) was higher in the non-responder group (Fig. 9B). GO enrichment analysis showed that B-cell-related pathways were significantly activated in the non-responder group (Fig. 9C), and the immune cell quantification algorithm results also showed that B cells were increased in the no-responder group compared with responder group (Fig. 9D).

**Fig. 10:**
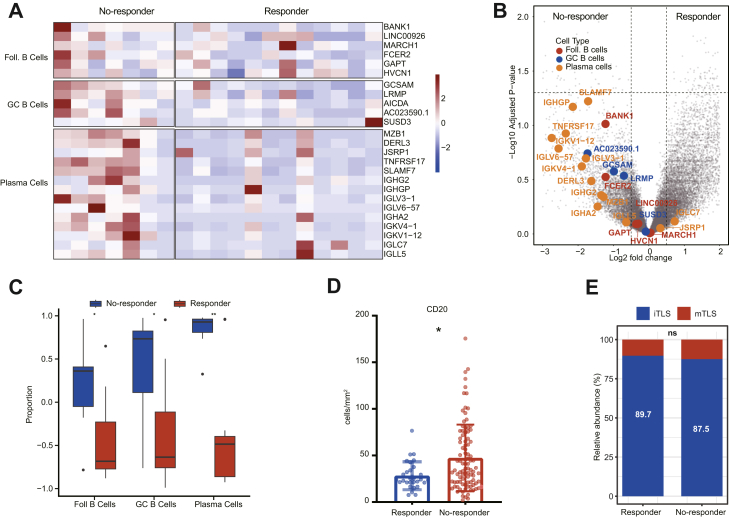
**Non-responder tumours are enriched for plasma-cell gene scores**. (A) Hierarchical cluster of the three B cell subset signatures. Samples are ordered by responder and non-responder (n = 19). (B) Volcano plot representing differentially expressed genes between tumours with responder and non-responder. Genes from the three B-cell signatures are highlighted. (C) Boxplots depicting signature Z scores for the plasma cell, GC B cell, and follicular B cells, grouped by responder and non-responder. Wilcoxon P values are reported. P values were derived from the Mann–Whitney U tests. (D) Box plots depict the density of CD20^+^ B cells, grouped by responder and non-responder. Wilcoxon P values are reported. P values were derived from the Mann–Whitney U tests. (E) The expression ratio of mTLS in post-neoTx specimens was compared in responder and non-responder. P values were derived from the χ2 test. ∗P < 0.05, ∗∗P < 0.01, ∗∗∗P < 0.001, ∗∗∗∗P < 0.0001.

No significant differences in plasma cell-, GC B cell-, and follicular B cell-associated gene expression were observed between the responder and non-responder group (Fig. 10A and B). Further enrichment analysis confirmed that, the non-responder group exhibited a higher enrichment score in plasma cell– (P < 0.01, Mann–Whitney U test), GC B cell– (P < 0.05, Mann–Whitney U test), and follicular B cell–associated gene signatures (P < 0.05, Mann–Whitney U test) compared with the responder group (Fig. 10C). In the IHC cohort (n = 125), the density of CD20^+^ B cell was significantly higher in the no-responder group compared with the responder group (P = 0.023, Mann–Whitney U tests) (Fig. 10D). However, despite these differences in B cell populations, no significant variation in TLS maturation was detected between the two groups (Fig. 10E). These findings raise the possibility that patients who do not respond to neoTx exhibit enhanced B cell-mediated immune responses, though this requires further validation.

## Discussion

Our study expands current understanding of the role of TLS in the TIME, particularly in LARC. We demonstrate that mTLS are associated with increased infiltration of B cells and plasma cells, as well as improved clinical outcomes. These findings support the notion that mTLS contribute to the effective B cell-mediated antitumour immunity. Furthermore, compared with NT group, neoTx group exhibited increased T cell infiltration and reduced mTLS. These findings suggest differential effects on distinct arms of the adaptive immune system. Interestingly, B cell infiltration and plasma cell activity were still observed in the non-responder group, pointing to a potentially compensatory, TLS-independent humoural immune response.

TLSs are organised lymphoid aggregates comprising a network of specialised fibroblasts that share both functional and structural characteristics with SLOs, particularly lymph nodes.^3^ For example, both TLS and SLOs can drive antigen-specific immune responses and possess specialised blood vessels known as high endothelial venules, which facilitate lymphocyte transmigration from the blood into lymphoid tissues.^7^ We categorised patients into mTLS and iTLS groups using H&E and mIF. Our study demonstrated a significant enrichment of plasma cell and B cell subsets in mTLS group compared with iTLS group. The presence of mTLS is positively associated with the activation of humoural immune pathways and shows significant correlation with increased expression of genes related to B cell differentiation and plasma cell function. Importantly, our data provide evidence that TLS maturation is associated with distinct plasma cell subtypes, characterised by higher levels of IgG^+^ and IgA^+^ plasma cells in mTLS tumours, thereby establishing a link between TLS structural maturity and antibody class distribution within the TME. Enhanced humoural immunoreactivity in mTLS is further evidenced by increased IgG and IgA expression. Consistent with our findings, Zhang et al. reported that mTLS tumours are enriched with IgG^+^ plasma cells and are associated with improved clinical outcomes. However, they observed that iTLS tumours are characterised by IgA^+^ plasma cells.^29^ Therefore, further studies are needed to investigate the effects of mTLS on different antibody production and their actions. Similarly, our data indicate that mTLS is closely associated with increased intratumoural B cell infiltration, particularly plasma cells, GC B cells and follicular B cells. This suggests that mTLS with functional GC plays a crucial role in orchestrating a robust B cell-mediated antitumour response.^6^^,^^12^^,^^28^^,^^30^^,^^31^ Furthermore, the correlation between high intratumoural plasma cell infiltration and improved patient prognosis underscores the potential of plasma cells as favourable prognostic markers in LARC. These findings highlight the critical role of mTLS in fostering a protective antitumour immune environment.

NeoTx is the standard treatment for patients with LARC (13). Recent studies have demonstrated that neoTx can significantly modulate the TIME. For example, neoTx has been shown to increase cytotoxic T cells, tissue-resident memory T cells, and B cells.^32^ Consistent with these findings, our study demonstrated that neoTx group exhibited enhanced T-cell-mediated cytotoxic responses, as evidenced by the enrichment of CD8^+^ T cells and cytotoxic T-cell-associated genes. In contrast, our study revealed that neoTx did not enhance B-cell-associated immunity and instead reduced TLS maturation. Similarly, Rupp et al. reported that neoTx is associated with suppression of the B cell-centred immune landscape in pancreatic ductal adenocarcinoma.^33^ This suppression is evidenced by decreased frequencies of pan B cells, GC B cells, plasmablasts, and plasma cells, along with a reduced abundance of TLS and the downregulation of major B cell pathways.^33^ These observations suggest that neoTx preferentially promotes T-cell-mediated cytotoxic immunity, potentially through the induction of immunogenic cell death and the release of tumour antigens that activate T cells. In contrast, the maturation of TLS, which is critical for effective B-cell activation and humoural responses, may be more sensitive to neoTx-induced tissue remodelling or stromal alterations. Consequently, while neoTx enhances T-cell infiltration and function, its impact on B-cell-mediated immunity appears limited, likely due to impaired TLS formation and organisation.

Our analysis revealed no significant association between neoTx sensitivity and TLS maturation, likely due to the near-complete absence of mTLS in neoTx group. This finding is consistent with our previous research, which demonstrated that neoTx significantly reduces both TLS density and maturation.^20^ Several factors may contribute to this phenomenon. First, the formation and maintenance of TLS require a sustained tumour burden,^5^ which neoTx may disrupt. Second, mature TLSs—particularly those with GC—are highly proliferative structures susceptible to damage from chemoradiotherapy.^34^ Third, TLS maturation is a time-dependent process; the chronic nature of human tumours provides sufficient time for TLS development, whereas TLSs typically do not form in rapidly growing murine tumours.^11^ Finally, the interval between neoTx and surgery may be too short to permit TLS reformation.

Although neoTx is associated with a reduction in mTLS, we observed that B-cell–mediated immune responses remained elevated in non-responders. This observation could indicate that B cell activation and recruitment might occur through mTLS-independent mechanisms. Several such pathways could potentially sustain B cell activity in this setting. First, activation may take place in SLO, such as draining lymph nodes, where tumour antigens released following neoTx are presented to naïve B cells. The therapy-induced alteration in antigen release may favour a B-cell response in SLOs that is decoupled from the intratumoural TLS niche. Second, poorly organised lymphoid aggregates or stromal networks within the tumour—which do not meet the strict histological criteria for TLS—may still provide sufficient co-stimulatory signals to support B cell proliferation and differentiation. Third, innate immune signals, such as toll-like receptor (TLR) ligands released from therapy-damaged cells, could directly activate B cells in a T-cell–independent manner, potentially leading to a more polyclonal and less tumour-specific antibody response. These possibilities raise the intriguing hypothesis that non-responders, who exhibit a B-cell–enriched microenvironment, may be candidates for strategies that further enhance humoural immunity—such as cancer vaccines or immunomodulators that foster lymphoid organisation—or, alternatively, for approaches that target specific B-cell subsets if the response is deemed non-productive. Further mechanistic studies are needed to clarify the functional roles and antigen specificity of TLS-independent B cell infiltration, which may inform novel combinatorial strategies to overcome resistance to neoTx.

This study has several limitations. First, scRNA-seq and paired scBCR-seq analyses were performed on a limited number of tumour samples, which may affect the generalisability of these findings and warrants validation in larger cohorts. Second, this was a retrospective study, which may be subject to selection bias and incomplete clinical data. Further validation by researchers from other groups is necessary to confirm our findings. Third, as a cross-sectional study, causal or temporal relationships between neoTx and TLS maturation cannot be established. Assessment of TLS dynamics is further limited because pre-treatment biopsies are often small and sample the tumour centre, whereas TLS are primarily located at the invasive margin. Fourth, while we validated the prognostic value of a plasma cell signature and TLS signature in public rectal cancer cohorts, the lack of large, publicly available datasets for patients with LARC who received neoTx limited our ability to externally validate the therapy-specific findings related to TLS disruption and B-cell responses in non-responders. Future multi-institutional prospective studies are warranted to confirm these observations. Nevertheless, our analysis included a relatively large number of cases from multiple centres, enhancing the robustness of our cross-sectional observations. Future prospective studies with optimised sampling strategies will be important to validate TLS evolution in response to therapy.

In conclusion, our study highlights the critical role of mTLS in orchestrating robust B cell-centred antitumour immune responses. mTLS were associated with enhanced B cell and plasma cell activity. Additionally, neoTx significantly reshaped the intratumoural immune landscape, activating a T-cell-mediated response. Further subgroup analysis revealed that certain patients might exhibit resistance to neoTx; Interestingly, these patients showed enriched B cell responses, which could hypothetically render them more amenable to combined neoTx and immunotherapy. These findings provide valuable insights into the underlying mechanisms of tumour biology and treatment outcomes in RC, proposing potential therapeutic strategies that target both B cell- and T cell-mediated responses. Future studies should explore approaches to promote TLS maturation and remodel the intratumoural immune landscape, with the aim of improving treatment outcomes in RC.

## Contributors

N.T., Q.Y.W., and Y.L. contributed equally to this work. Conceptualisation: N.T., Q.Y.W., Y.L., J.F.D; Data curation: N.T., Q.Y.W., Y.L., W.T.Z., W.X.L, H.Y.C., L.S., Q.Y., X.D., J.H.D., J.C.B; Funding acquisition: J.F.D; Methodology: N.T., Q.Y.W., W.T.Z., W.X.L, R.A., H.Y.Z., A.J.L; Resources: J.F.D; Software: J.F.D; Visualisation: N.T., Q.Y.W., W.T.Z., R.A., H.Y.Z., A.J.L; Supervision: G.C., B.W., J.F.D; Validation: Y.L., H.Y.C; Writing—original draft: N.T., Q.Y.W; Writing—review & editing: W.T.Z., W.X.L, H.Y.C., R.A., H.Y.Z., L.S., Q.Y., X.D., J.H.D., J.C.B., G.C., B.W., J.F.D; N.T., Q.Y.W., Y.L., and J.F.D. accessed and verified the underlying data. All authors approved the submission of the final manuscript.

## Data sharing statement

All sequencing data generated in this study have been deposited in the Genome Sequence Archive. The scRNA-seq data are available under accession number HRA012259, the bulk mRNA-seq data under HRA012500. All other data supporting the findings of this study are included in the article and its supplementary information files.

## Declaration of interests

The authors declare that they have no conflict of interest.

## Appendix A Supplementary data

Supplemental Figure S1 mTLS tumours enriched for B-cell gene scores in the PUMCH cohort. (A) Hierarchical cluster of the T and B cell subset signatures. Samples are ordered by mTLS and iTLS (n = 67). (B) Volcano plot representing differentially expressed genes between tumours with mTLS and iTLS. Genes from the T and B cell subset signatures are highlighted. (C) GO and KEGG enrichment analysis between the tumour with mTLS and iTLS. (D) Immune cell infiltration differences between mTLS and iTLS tumours were evaluated using computational tools, including CIBERSORT, XCELL, MCPCOUNTER, and QUANTISEQ. P values were derived from the Mann–Whitney U test. ∗P < 0.05, ∗∗P < 0.01, ∗∗∗P < 0.001, ∗∗∗∗P < 0.0001.

Supplemental Figure S2 mTLS tumours enriched for plasma-cell gene scores in the PUMCH cohort. (A) Hierarchical cluster of the three B-cell subset signatures. Samples ordered by mTLS and iTLS (n = 67). (B) Volcano plot representing differentially expressed genes between tumours with mTLS and iTLS. Genes from the three B-cell signatures are highlighted. (C) Boxplots depicting signature Z scores for the plasma cells, GC B cells, and follicular B cells, grouped by mTLS and iTLS status. P values were derived from the Mann–Whitney U test. ∗P < 0.05, ∗∗P < 0.01, ∗∗∗P < 0.001, ∗∗∗∗P < 0.0001.

Supplementary Tables

Analysis settings
