# Supplementary figures and images for: Mature tertiary lymphoid structures support B cell-mediated antitumour immunity and are disrupted by neoadjuvant therapy in rectal cancer: a multicentre, retrospective study
Source: eBioMedicine. 2025 Nov 19;122:106030. doi: 10.1016/j.ebiom.2025.106030 (PMC12670569; doi:10.1016/j.ebiom.2025.106030)

**A**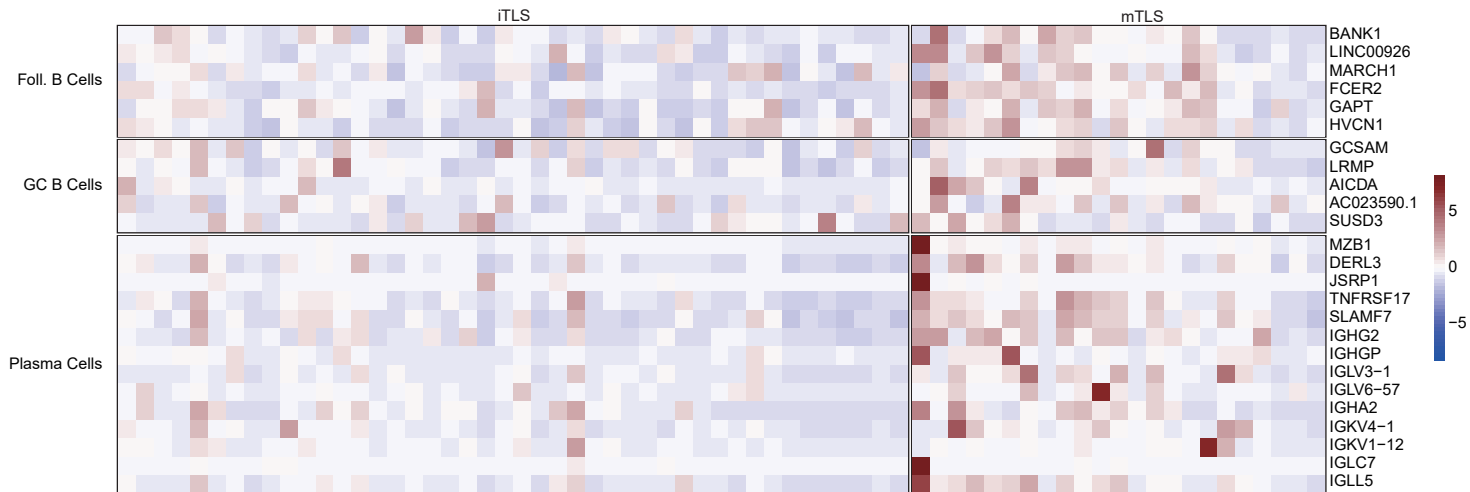**B**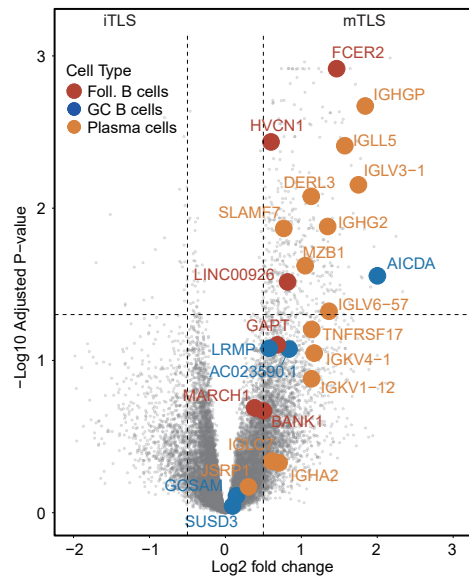**C**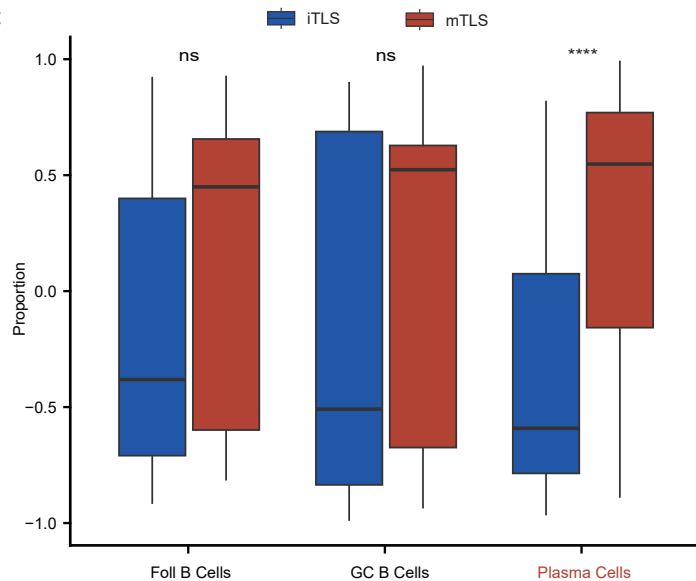

Supplement: Supplemental Figure S2 — mTLS tumours enriched for plasma-cell gene scores in the PUMCH cohort. (A) Hierarchical cluster of the three B-cell subset signatures. Samples ordered by mTLS and iTLS (n = 67). (B) Volcano plot representing differentially expressed genes between tumours with mTLS and iTLS. Genes from the three B-cell signatures are highlighted. (C) Boxplots depicting signature Z scores for the plasma cells, GC B cells, and follicular B cells, grouped by mTLS and iTLS status. P values were derived from the Mann–Whitney U test. ∗P < 0.05, ∗∗P < 0.01, ∗∗∗P < 0.001, ∗∗∗∗P < 0.0001. [file mmc2.pdf]
