# Supplementary material for: Mature tertiary lymphoid structures support B cell-mediated antitumour immunity and are disrupted by neoadjuvant therapy in rectal cancer: a multicentre, retrospective study
Source: eBioMedicine. 2025 Nov 19;122:106030. doi: 10.1016/j.ebiom.2025.106030 (PMC12670569; doi:10.1016/j.ebiom.2025.106030)
Supplement: Supplemental Figure S1 — mTLS tumours enriched for B-cell gene scores in the PUMCH cohort. (A) Hierarchical cluster of the T and B cell subset signatures. Samples are ordered by mTLS and iTLS (n = 67). (B) Volcano plot representing differentially expressed genes between tumours with mTLS and iTLS. Genes from the T and B cell subset signatures are highlighted. (C) GO and KEGG enrichment analysis between the tumour with mTLS and iTLS. (D) Immune cell infiltration differences between mTLS and iTLS tumours were evaluated using computational tools, including CIBERSORT, XCELL, MCPCOUNTER, and QUANTISEQ. P values were derived from the Mann–Whitney U test. ∗P < 0.05, ∗∗P < 0.01, ∗∗∗P < 0.001, ∗∗∗∗P < 0.0001. [file mmc1.pdf]

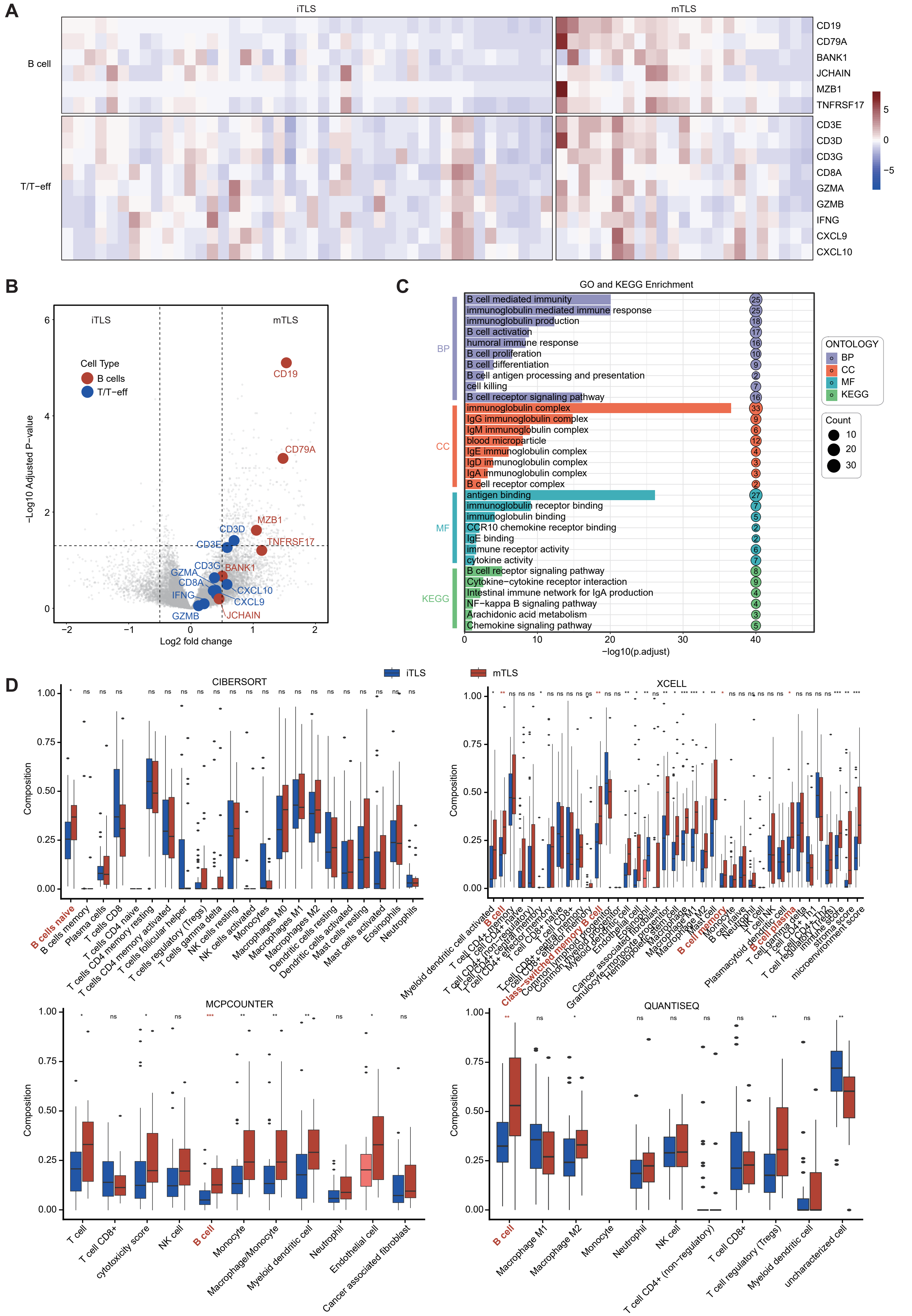
A
iTLS
mTLS
B cell
T/T−eff
CD19
CD79A
BANK1
JCHAIN
MZB1
TNFRSF17
CD3E
CD3D
CD3G
CD8A
GZMA
GZMB
IFNG
CXCL9
CXCL10
5
0
−5
B
iTLS
mTLS
Cell Type
B cells
T/T−eff
−Log10 Adjusted P−value
Log2 fold change
CD19
CD79A
MZB1
TNFRSF17
CD3D
CD3E
CD3G
BANK1
GZMA
CD8A
IFNG
GZMB
CXCL10
CXCL9
JCHAIN
C
GO and KEGG Enrichment
BP
CC
MF
KEGG
B cell mediated immunity
immunoglobulin mediated immune response
immunoglobulin production
B cell activation
humoral immune response
B cell proliferation
B cell differentiation
B cell antigen processing and presentation
cell killing
B cell receptor signaling pathway
immunoglobulin complex
IgG immunoglobulin complex
IgM immunoglobulin complex
blood microparticle
IgE immunoglobulin complex
IgD immunoglobulin complex
IgA immunoglobulin complex
B cell receptor complex
antigen binding
immunoglobulin receptor binding
immunoglobulin binding
CCR10 chemokine receptor binding
IgE binding
immune receptor activity
cytokine activity
B cell receptor signaling pathway
Cytokine−cytokine receptor interaction
Intestinal immune network for IgA production
NF−kappa B signaling pathway
Arachidonic acid metabolism
Chemokine signaling pathway
−log10(p.adjust)
ONTOLOGY
BP
CC
MF
KEGG
Count
10
20
30
D
iTLS
mTLS
CIBERSORT
Composition
B cells naive
B cells memory
Plasma cells
T cells CD8
T cells CD4 naive
T cells CD4 memory resting
T cells CD4 memory activated
T cells follicular helper
T cells regulatory (Tregs)
T cells gamma delta
NK cells resting
NK cells activated
Monocytes
Macrophages M0
Macrophages M1
Macrophages M2
Dendritic cells resting
Dendritic cells activated
Mast cells resting
Mast cells activated
Eosinophils
Neutrophils
XCELL
Myeloid dendritic cell activated
B cell
T cell CD4+ memory
T cell CD4+ naive
T cell CD4+ (non−regulatory)
T cell CD4+ central memory
T cell CD4+ effector memory
T cell CD8+ naive
T cell CD8+
T cell CD8+ central memory
T cell CD8+ effector memory
Class−switched memory B cell
Common lymphoid progenitor
Common myeloid progenitor
Myeloid dendritic cell
Endothelial cell
Eosinophil
Cancer associated fibroblast
Granulocyte−monocyte progenitor
Hematopoietic stem cell
Macrophage
Macrophage M1
Macrophage M2
Mast cell
B cell memory
Monocyte
B cell naive
Neutrophil
NK cell
T cell NK
Plasmacytoid dendritic cell
B cell plasma
T cell gamma delta
T cell CD4+ Th1
T cell CD4+ Th2
T cell regulatory (Tregs)
immune score
stroma score
microenvironment score
MCPCOUNTER
T cell
T cell CD8+
cytotoxicity score
NK cell
B cell
Monocyte
Macrophage/Monocyte
Myeloid dendritic cell
Neutrophil
Endothelial cell
Cancer associated fibroblast
QUANTISEQ
B cell
Macrophage M1
Macrophage M2
Monocyte
Neutrophil
NK cell
T cell CD4+ (non−regulatory)
T cell CD8+
T cell regulatory (Tregs)
Myeloid dendritic cell
uncharacterized cell
